# Methylation Landscape: Targeting Writer or Eraser to Discover Anti-Cancer Drug

**DOI:** 10.3389/fphar.2021.690057

**Published:** 2021-06-03

**Authors:** Wen-min Zhou, Bin Liu, Amin Shavandi, Lu Li, Hang Song, Jian-ye Zhang

**Affiliations:** ^1^ Key Laboratory of Molecular Target & Clinical Pharmacology and the State Key Laboratory of Respiratory Disease, School of Pharmaceutical Sciences & the Fifth Affiliated Hospital, Guangzhou Medical University, Guangzhou, China; ^2^ Department of Cellular and Molecular Biology, Beijing Chest Hospital, Capital Medical University/Beijing Tuberculosis and Thoracic Tumor Research Institute, Beijing, China; ^3^ BioMatter Unit, École Polytechnique de Bruxelles, Université Libre de Bruxelles (ULB), Brussels, Belgium; ^4^ Department of Biochemistry and Molecular Biology, School of Integrated Chinese and Western Medicine, Anhui University of Chinese Medicine, Hefei, China

**Keywords:** epigenetics, writers, readers, erasers, cancer, epigenetic drugs

## Abstract

Cancer is a major global health challenge for our health system, despite the important pharmacological and therapeutic discoveries we have seen since past 5 decades. The increasing prevalence and mortality of cancer may be closely related to smoking, exposure to environmental pollution, dietary and genetic factors. Despite significant promising discoveries and developments such as cell and biotechnological therapies a new breakthrough in the medical field is needed to develop specific and effective drugs for cancer treatment. On the development of cell therapies, anti-tumor vaccines, and new biotechnological drugs that have already shown promising effects in preclinical studies. With the continuous enrichment and development of chromatin immunoprecipitation sequencing (ChIP-seq) and its derivative technologies, epigenetic modification has gradually become a research hotspot. As key ingredients of epigenetic modification, Writers, Readers, Erasers have been gradually unveiled. Cancer has been associated with epigenetic modification especially methylation and therefore different epigenetic drugs have been developed and some of those are already undergoing clinical phase I or phase II trials, and it is believed that these drugs will certainly assist the treatment in the near future. With respect to this, an overview of anti-tumor drugs targeting modified enzymes and de-modified enzymes will be performed in order to contribute to future research.

## Introduction

The epigenetic modification will affect gene expression. The genetic changes in gene expression or cell phenotype can happen without the sequence change of DNA. The phenomenon is called the epigenetic phenomenon, which can be stably inherited and may be reversible during embryonic development and cell proliferation (Harvey et al., 2018). The core of epigenetics includes various covalent modifications of histones and nucleic acids, which coordinately regulate chromatin structure and gene expression. Disorders of the epigenetic processes will drive abnormal transcription programs and promote the occurrence and progression of cancer (Sapienza and Issa, 2016; Nebbioso et al., 2018). According to the current research results, the regulatory mechanisms of epigenetic modification mainly include DNA methylation (Skvortsova et al., 2019), RNA interference (Holoch and Moazed, 2015), histone modification (Stoll et al., 2018), chromatin remodeling (Dawson and Kouzarides, 2012), and nucleosome localization (Allis and Jenuwein, 2016).

Currently, DNA methylation is the most fully studied form of epigenetic modification, which mainly involves adding a methyl group to the C5 position of the cytosine base to produce 5-methylcytosine. Normal methylation is necessary for maintaining cell growth and metabolism, while abnormal DNA methylation can cause diseases (such as cancers). The reason for the occurrence of this event may be that, on the one hand, abnormal methylation may prevent the transcription of tumor suppressor genes, and on the other hand, it may cause genome instability. Therefore, the study of DNA methylation is very helpful for understanding biological growth and disease treatment (Koch et al., 2018). In the cell nucleus, DNA is wrapped around histones and packaged to form a chromatin structure, and the tightness of chromatin packaging determines the activity of gene expression. Studies have confirmed that chromatin can switch between the “on and off” states by regulating the chemical modification of histones (Bannister and Kouzarides, 2011; Calo and Wysocka, 2013). Non-coding RNA refers to a functional RNA molecule that cannot be translated into protein and has a regulatory effect, and it plays a very important role in regulating gene expression. The regulation of non-coding RNA is the regulation of gene transcription through certain mechanisms, such as RNA interference (Holoch and Moazed, 2015).

Chromatin remodeling involves a series of biological processes mediated by the chromatin remodeling complex, which is characterized by nucleosome changes in chromatin, and it was considered to be an important epigenetic mechanism (Nacev et al., 2020). Studies have suggested that nucleosomes were barriers to gene transcription, and DNA tightly wound on histones cannot bind to many transcription factors and activation factors. In each type of cell, several specific genes are activated while others inhibited; thus, resulting in a variety of gene expression patterns. Therefore, changes in the position of nucleosomes in the genome have an important impact on regulating gene expression. With the development of life cycle activities, such as DNA replication, recombination, repair, and transcriptional regulation, the positioning of nucleosomes in chromatin has undergone dynamic changes. This continuous change requires participation of a series of chromatin remodeling complexes (Dawson and Kouzarides, 2012).

**FIGURE 1:**
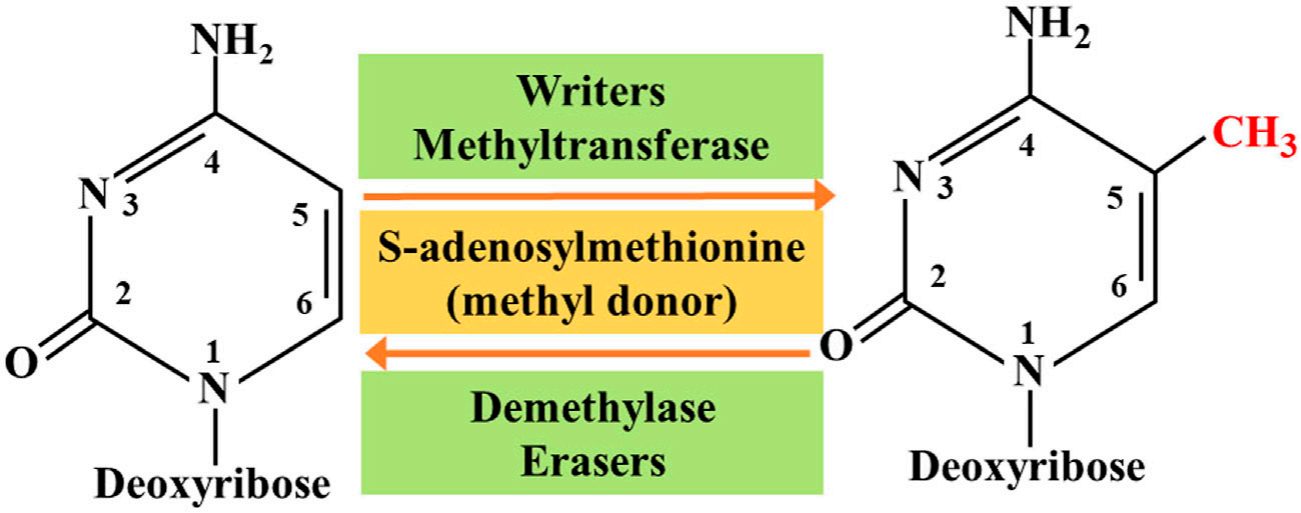
Methylation interactions between writers and erasers.

So far, researchers have identified four different types of cytosine residue modifications in DNA, including methylation, hydroxymethylation, formylation, and carboxylation (Biswas and Rao, 2018). In addition, more than ten different types of histone modifications have been identified; and among them, methylation and acetylation are the most stable and suitable. With continuous progress in the research on epigenetics, the key roles associated with these modifications are gradually being deciphered. They are as follows: 1) the writers-a large number of enzymes that can modify nucleotide bases and specific amino acid residues in histones, 2) the erasers-a group of enzymes proficient in removing these markers, and 3) the readers -a series of different proteins that possess specific domains that can recognize specific epigenetic marks in a locus. Together, these enzymes and protein domains constitute epigenetic tools (Biswas and Rao, 2018; Zaccara et al., 2019). The modification of DNA and histones occurs by adding various chemical groups using a variety of enzymes. We will focus on the most extensive study of epigenetic modifications, namely methylation. Both DNA and histones are prone to methylation, and this modification usually controls gene expression in cells through changes in transcription activation or inhibition. Here, the epigenetic writers, which we intend to focus on include DNA methyltransferase, histone lysine methyltransferase, and protein arginine methyltransferase (Biswas and Rao, 2018). Depending on the requirements of the cell to modify the expression state of the locus, epigenetic marks formed by histone post-translational modification and DNA covalent modification can be removed. To achieve this purpose, a group of enzymes called erasers can counter the writer's activities. The eraser can catalyze the removal of epigenetic marks, thereby reducing the effect of epigenetic marks on transcription; thus, leading to regulation of gene expression. The key difference between genetic mutation and epigenetic modification is that epigenetic changes are reversible, which makes them attractive drug targets. On the whole, the ultimate goal of research on tumor-related epigenetic mechanisms is to apply them to clinical diagnosis and preventive treatment (Figure 1).

## Writers

Modifications of DNA and histone proteins occur through the additions of various chemical groups utilizing numerous enzymes. Although a plethora of modifications are possible, we have focused on the two most widely studied epigenetic alterations, methylation and acetylation. Both DNA and histone proteins are prone to methylation, while acetylation is associated only with histones. These two modifications frequently govern the gene expression pattern in a cell by switching between transcriptional activation or repression. Changes in global DNA methylation and individual gene methylation patterns are distinguishing features of cancer cells, which are governed by DNMT: DNA methyltransferase. Global DNA hypomethylation and hypermethylation of the promoter regions of tumor suppressor genes have been reported in malignant cells (Kulis and Esteller, 2010). These modifications are caused by DNMT providing a viable target for developing drugs against these enzymes.

Abnormal DNA methylation at the C5 position of the cytosine catalyzed by DNMTs is not only related to silencing of many tumor suppressor genes, but also to other diseases. Small molecule inhibitors of DNMTs are the most widely used epigenetic therapies for cancer treatment, mainly for the treatment of myelodysplastic syndrome (MDS) and acute myeloid leukemia (AML) (Khan et al., 2013; Du et al., 2020). DNMT is usually overexpressed in various cancer tissues and cell lines. Since DNA methylation is reversible, DNMT is considered an important epigenetic target for drug development. DNMT inhibitors are categorized into nucleosides and non-nucleosides. Among these inhibitors, the nucleoside analogue azacytidine and its deoxy derivative decitabine are both irreversible DNMT inhibitors and have been approved for the treatment of bone marrow hyperplastic syndrome (Zhou et al., 2018b). Studies have shown that DNMTs can up-regulate the immune signal in ovarian cancer through the viral defense pathway (Chiappinelli et al., 2015).

Inhibitors of poly (ADP-ribose) polymerase 1 (PARP1) have shown promise for targeting cancer cells harboring mutations in the double-strand break (DSB) repair breast cancer genes, BRCA1 and BRCA2, where these drugs induce synthetic lethality. Studies have shown that PARP1 and DNMTs have shown an unexpected benefit in the combined treatment of non-small cell lung cancer (NSCLC). Chemoradiation is the main treatment method for NSCLC. Interestingly, combined treatment with PARP1 and DNMTs can make NSCLC cells very sensitive to ionizing radiation both *in vitro* and *in vivo*. With respect to the key mechanism, DNMTs may produce a BRCA-like phenotype by down-regulating the expression of key homologous recombination and non-homologous end joining genes (Abbotts et al., 2019).

The enhancer of Zeste homolog 2 (EZH2) is histone methyltransferase, and it catalyzes the methylation of histone 3 lysine 27, which is a sign of transcriptional inhibition. Many studies have clarified the complex role of EZH2 in normal biology and tumorigenesis. The effect of EZH2 on tumorigenesis is attributed to direct gene overexpression and point mutations leading to gain or loss of function. To a large extent, point mutations can increase the function of lymphoma and inactivate mutations in myeloid malignancies, and overexpression is the main manifestation of epithelial malignancies and specific lymphoma subtypes. Interestingly, EZH2 may have a dual function, and based on the dynamic expression of EZH2 in cell differentiation and cell cycle progression, it has the ability to act as both an oncogene and a tumor suppressor. Tazemetostat is the first EZH2 inhibitor, and it has shown enhanced clinical activity in mutant follicular lymphoma and diffuse large B-cell lymphoma. A new treatment strategy is required not only for the treatment of lymphoma, but it may also be beneficial in the treatment of many other malignant tumors (Lue and Amengual, 2018).

Another *in vivo* clinical study on tazemetostat published in 2018 reported that it showed good safety and anti-tumor activity in refractory B-cell non-Hodgkin’s lymphoma and advanced solid tumors, including epithelioid sarcoma. Further clinical studies of tazemetostat monotherapy are ongoing in phase 2 trials in adults and phase 1 trials in children. A total of 64 patients (21 cases of B-cell non-Hodgkin's lymphoma and 43 cases of advanced solid tumors) received treatment with tazemetostat. No treatment-related deaths occurred. A durable objective response was observed in 21 patients with B-cell non-Hodgkin's lymphoma (Italiano et al., 2018) (NCT01897571).

Excellent efficacy of EZH2 inhibitors in cancer is likely to cause the problem of drug resistance. Studies have confirmed that the role of Forkhead box transcription factor-1 (FOXO1) is linked to the role of EZH2 inhibitors in cancer. It is believed that the FOXO1 gene is a new inhibitory target of EZH2 and FOXO1 is a key mediator of EZH2 inhibition and induction of prostate cancer cell death. Phosphatase and tension homolog (PTEN) is a well-known tumor suppressor and FOXO1 is a key downstream effector of PTEN in inhibiting cell growth and survival. Further studies have proved that EZH2 inhibitors cannot effectively induce PTEN-deficient cancer cell death, but they can be overcome by combination therapy with taxanes. The following mechanism is likely: EZH2 inhibits FOXO1 expression and may serve as a target of EZH2 inhibitors, which can be used to overcome EZH2 inhibitor resistance in PTEN mutant cancers or to treat PTEN-deficient prostate cancer in combination with taxane. A new inhibitory target and FOXO1 is a key mediator of EZH2 inhibition to induce prostate cancer cell death. Further studies have proved that EZH2 inhibitors cannot effectively induce PTEN-deficient cancer cell death, but they can be overcome by combination therapy with taxanes (Ma et al., 2019). Furthermore, Bitler et al. believe that inhibition of EZH2 methyltransferase can lead to regression of ARID1A mutant tumors in mouse models of ovarian cancer. This is because PIK3IPI is the target of ARID1A and EZH2, which up-regulates PIK3IPI by inhibiting EZH2, and ultimately inhibits the oncogenic PI3K/Akt signal (Bitler et al., 2015).

DOT1L is the only known histone 3 lysine 79 (H3K79) methyltransferase, while AML or acute lymphocytic leukemia (ALL) is a malignant clonal disease of hematopoietic stem cells, and without any special treatment, these patients can only survive for about 3 months. Some of these patients can even die within a few days after the diagnosis. Both these diseases are associated with aberration in mixed lineage leukemia (MLL) gene (also known as KMT2A) translocation at chromosome 11q23, and both show a poor prognosis. If DOT1L can become an attractive target for the treatment of acute leukemia, it will be of great benefit to the medical field. Pinometostat (EPZ-5676) is a small molecule inhibitor of DOT1L histone methyltransferase interference. In an *in vivo* clinical trial, the DOT1L inhibitor pinometostat can reduce H3K79 methylation and has moderate clinical activity in adult acute leukemia. This study demonstrates the therapeutic potential of DOT1L in the mixed lineage leukemia (MLL) gene rearrangement of leukemia and lays the foundation for future combination therapy in this patient population (Stein et al., 2018) (NCT01684150).

In another study, convincing evidence was obtained by using DOT1L inhibitors as targeted therapy for MLL. EPZ004777 is a potent and selective inhibitor of DOT1L. According to the reports, MLL cells treated with EPZ004777 can selectively inhibit H3K79 methylation and can effectively block the expression of leukemia-associated genes. In addition, *in vitro* experiments showed that EPZ004777 has a selective killing effect on leukemia cells translocated by the MLL gene, and it has minimal effect on non-MLL translocated cells. *In vivo* experiments also showed a good performance; i.e., by administering EPZ004777 to a mouse xenograft model of MLL, the survival period can be significantly extended (Daigle et al., 2011).

It is undeniable that effective inhibitors of DOT1L have achieved many surprising results in targeting leukemia with MLL gene rearrangement. Research on this subject will continue in the future. In a document published in 2019, it has been confirmed that pinometostat as a DOT1L inhibitor has entered a phase 1 clinical trial to treat children with relapsed/refractory leukemia with MLL gene rearrangement Patient (NCT02141828). It has also been confirmed that pinometostat is undergoing a phase 1/2 clinical trial for evaluating the combination of pinometostat and standard chemotherapy for the treatment of newly diagnosed MLL rearranged leukemia in children and adults (NCT03724084) (Lonetti et al., 2019).

Different from the leukemia mechanism of MLL gene translocation, DOT1L is also believed to play an important role in the occurrence and development of breast cancer. Studies have suggested that DOT1L can target the gene expression of epithelial-mesenchymal transition (EMT) promoters by cooperating with the c-Myc/p300 transcriptional activity complex, thereby playing an important role in the occurrence and development of breast cancer, which suggests that DOT1L is a potential therapeutic target for invasive breast cancer (Lee and Kong, 2015).

Similarly, the new psammaplin A analogue (PsA-3091) is considered as a new template for DOT1L inhibitors, and it shows an effective inhibitory effect on DOT1L-mediated H3K79 methylation. As already known, triple-negative breast cancer (TNBC) is the most difficult disease to treat among women and has a high risk of metastasis. According to reports, PsA-3091 has a significant inhibitory effect on the proliferation, migration, and invasion of TNBC cells, and it can significantly enhance the expression of E-cadherin and inhibit the expression of N-cadherin, ZEB1, and vimentin. In addition, by developing an orthotopic mouse model, it has been suggested that PsA-3091 can effectively inhibit lung metastasis and tumor growth by regulating DOT1L activity and EMT biomarkers. *In vivo* and *in vitro* studies have confirmed that PsA-3091 exhibits excellent anti-tumor and anti-metastasis effects, and it can be speculated as a promising potential target for the treatment of patients with metastatic breast cancer (Byun et al., 2019). In addition, DOT1L inhibitors have also shown surprising performance in neuroblastoma (Wong et al., 2017) and colorectal cancer (Huang et al., 2017).

**FIGURE 2:**
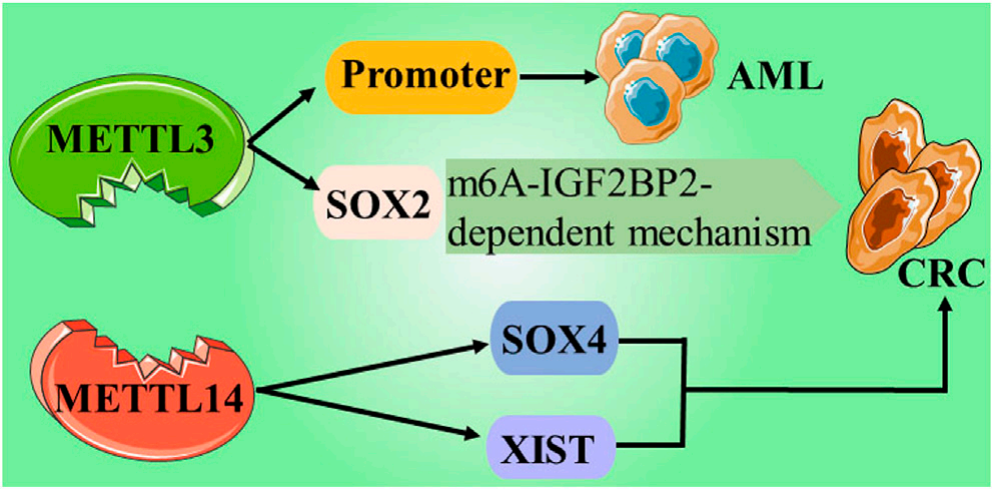
Give examples of the role and mechanisms involved writers in cancers.

In recent years, abundant research evidence has shown that PRMT5, as an oncogene, can play an indispensable regulatory role in the pathological progression of various human cancers by promoting the proliferation, invasion, and metastasis of cancer cells. This indicates that PRMT5 may become a potential biomarker or therapeutic target for cancer. Regulation of epigenetics may be one of the main mechanisms by which PRMTs affect cell activities. Currently, PRMT1 and PRMT5 inhibitors have entered the first phase of clinical trials; thus, opening up a new path for the treatment of solid tumors and hematological malignancies (Jarrold and Davies, 2019) (NCT03666988) (Table 1 and Figure 2).

**TABLE 1:** Describe the author and eraser mechanism in cancer.

Component	Disease	Target	Function	Regulation	Refs
METTL3	AML	Promoter	Writers	Downregulation of it leads to cell cycle arrest and leukemia cell differentiation	Barbieri et al. (2017)
METTL3	CRC	SOX2	Writers	Promote the progression of CRC through m6A-IGF2BP2-dependent mechanism	Li et al. (2019b)
METTL14	CRC	XIST	Writers	Inhibit the proliferation and metastasis of CRC by down-regulating XIST	Yang et al. (2020)
METTL14	CRC	SOX4	Writers	Inhibit the migration, invasion and metastasis of CRC cells through SOX4	Chen et al. (2020b)
WTAP	HCC	ETS1	Writers	Promotes the progression of HCC through m6A-HuR-dependent epigenetic silencing of ETS1	Chen et al. (2019)
DNMT	CML	MNC bone marrow	Writers	The expression level of DNMT mRNA is related to the disease progression of CML.	Li et al. (2015)
DNMT1	GC	PCDH10	Writers	By interacting with HOTAIR and miR-148b, it leads to the methylation of PCDH10, thereby promoting the development of GC	Seo et al. (2021)
DNMT1	HCC	miR-148a-3p	Writers	Block the negative regulation between miR-148a-3p, thereby inhibiting the stem cell characteristics of HCC cells	Li et al. (2020)
EZH2	PTEN-mutated cancer	FOXO1	Writers	Inhibition of FOXO1 can treat PTEN-proficient cancers	Ma et al. (2019)
EZH2	EOC	PRMT4	Writers	The activity of EZH2 determines the level of PRMT4 expression in EOC	Karakashev et al. (2018)
WTAP	SaOS	HMBOX1	Writers	Inhibit the expression of HMBOX1 in an m6A-dependent manner to promote the occurrence of SaOS.	Chen et al. (2020a)
FTO	AML	FB23 FB23–2	Erasers	Inhibit proliferation and promote differentiation/apoptosis of AML cell lines	Huang et al. (2019)
FTO	Leuke-mia	LILRB4	Erasers	Inhibit the maintenance and immune escape of cancer stem cells	Su et al. (2020)
FTO	NSCLC	USP7	Erasers	Promote the growth of NSCLC by regulating the m6A level of USP7 mRNA	Li et al. (2019a)
FTO	Cervical cancer	E2F1 Myc	Erasers	Overexpression of E2F1 or Myc can make up for the lack of FTO, thereby reducing cell proliferation and migration	Zou et al. (2019)
FTO	Breast tumor	BNIP3	Erasers	Promote breast tumor progression by inhibiting BNIP3	Niu et al. (2019)
ALKBH2	Bladder cancer	MUC1	Erasers	Promote the development of bladder cancer by regulating the expression of MUC1	Fujii et al. (2013)
ALKBH5	EOC	MiR-7 BCL-2	Erasers	Inhibition of autophagy in epithelial ovarian cancer through miR-7 and BCL-2	Zhu et al. (2019)
ALKBH5	PC	PER1	Erasers	Prevents the progression of pancreatic cancer through the post-transcriptional activation of PER1 that depends on m6A-YTHDF2	Guo et al. (2020)
ALKBH5	AML	LSCs LICs	Erasers	Selectively promote tumorigenesis and cancer stem cell self-renewal in AML	Shen et al. (2020)

METTL3, methyltransferase-like 3; CRC, Colorectal carcinoma; SOX2, sex determining region Y-box 2; XIST, X inactivate-specific transcript; SOX4, SRY-related high-mobility-group box; WTAP, Wilms tumor 1-associated protein; HCC, hepatocellular carcinoma; ETS1, ETS proto-oncogene 1; SaOS, osteosarcoma; CML, Chronic Myeloid Leukemia; PCDH10, Protocadherin 10; FOXO1, Forkhead box transcription factor-1; PRMT, protein arginine methyltransferase; EOC, Epithelial ovarian cancer; NSCLC, non-small cell lung cancer; Erasers, ubiquitin-specific protease; EOC, epithelial ovarian cancer; LSCs/LICs, leukemia stem/initiating cells.

## Erasers

In recent years, the regulatory role of epigenetic modifications in the occurrence and development of malignant tumors has received extensive attention. It is obvious that some epigenetic markers, whether in the form of post-translational modifications of histones or equivalent modifications of DNA, have proven to be non-permanent; i.e., removable. An enzyme called eraser appears immediately, which can remove these genetic markers, thereby resisting the writer's activity and leading to the regulation of gene expression (Biswas and Rao, 2018).

In the past few decades, with the rise of RNA epigenetics, the focus of N6-methyladenosine (m6A) RNA modification has gradually become a research hotspot in the field of biological sciences. In the early days, due to the incompatibility of technology and information, research on epigenetic modification was focused on the DNA and protein levels. Currently, the RNA epigenetic modification based on m6A has dynamic and reversible characteristics, and it is the most common mRNA epigenetic modification that exists in all higher eukaryotes. It involves fine regulation of many complex cellular processes, such as RNA processing, transportation, localization, translation, and degradation. Studies have shown that m6A can dynamically regulate RNA transport, localization, translation, and degradation through the abnormal expression of “writing”, “erasing”, and “reading” related factors. It can play a role in tumor development through a variety of mechanisms, including promotion or inhibition. Here, we mainly introduce the eraser, which can encode m6A demethylase to remove the m6A modification in the RNA molecule, which is the key to the reversibility of the m6A modification process (Roundtree et al., 2017). Currently, the identified “erasers” are mainly fat mass and obesity associated (FTO) protein and AlkB homolog 5 (ALKBH5) (Zou et al., 2016; Zhang et al., 2017). Although these two erasers have similar functions, they have different processes.

Studies have proved that FTO is not only limited to playing a major role in obesity-related diseases, but is also involved in the occurrence, development, and prognosis of a variety of cancers, including AML, glioblastoma, and breast cancer (Chen and Du, 2019; Lan et al., 2020). Long-term studies have shown that m6A modification is related to tumorigenesis, proliferation, invasion, and metastasis, and it acts as an oncogene or tumor suppressor gene in malignant tumors (Sun et al., 2019). Rhein, meclofenamic acid (MA), MO-I-500, fluorescein, and R-2-hydroxyglutarate (R-2HG) belong to a group of specific or non-specific FTO inhibitors that have been identified. Briefly, these small molecules are limited in their clinical applications due to their weak biological functions and low sensitivity and/or specificity (Huang et al., 2019).

Su et al. developed two highly effective FTO inhibitors, CS1 and CS2. Compared with the two previously reported FTO inhibitors (FB23–2 and MO-I-500), CS1 and CS2 showed better performance in inhibiting the viability of AML cells. Su et al. obtained a set of up- or down-regulated pathways through global gene set enrichment analysis (GSEA). Surprisingly, in the up-regulated pathway, CS1, CS2, and FTO shared most of the abundant signaling pathways and core genes. Among the down-regulated pathways, the pathways inhibited by FTO are also widely present in the pathways inhibited by CS1 or CS2. The results showed that CS1 and CS2 may be able to exhibit high-efficiency performance by regulating the basic signal pathway of FTO. Interestingly, further *in vivo* experiments showed that CS2 treatment significantly reduced leukemic infiltration and doubled overall survival. However, CS1 treatment did not exert any significant effect. Further analysis showed that poor solubility and absorption of CS1 may be the reason for its inability to function in the body, and Su et al. subsequently solved this problem. However, it is undeniable that the FTO inhibitors CS1 and CS2 with anti-tumor and low side effects derived from the data have great potential in clinical applications (Su et al., 2020). Su et al. also found that R-2HG can display anti-tumor activity by targeting the FTO/m6A/MYC/CEPPA signaling pathway. In terms of the mechanism, R-2HG inhibits the increase in fat mass and the expression of FTO, thereby increasing the modification of m6A RNA in leukemia cells sensitive to R-2HG; thus, leading to further reduction in the stability of MYC/CEPPA transcripts, and ultimately suppressing the related pathways (Su et al., 2018).

It is recognized that ubiquitin-specific protease 7 (USP7) has a wide range of substrates, and most of the substrates, such as p53, PTEN, and FOXO4, are related to tumor suppression, DNA repair, or immune response. Therefore, USP7 is a potential cancer treatment target. Studies have found that FTO can promote the expression of USP7 through demethylation and can increase the stability of USP7, which is expected to become a potential target for the treatment of human lung cancer (Li J. et al., 2019). FTO can directly interact with oncogenic factors E2F1 and Myc. Experiments showed that by inhibiting FTO, the translation function of the two factors can be disrupted, thereby inhibiting the proliferation and migration of cervical cancer cells. But the study also revealed an interesting point, i.e., the overexpression of oncogenic factors E2F1 and Myc can also counteract the proliferation or migration of cervical cancer cells induced by the lack of FTO, and weaken its inhibitory effect (Zou et al., 2019). Similarly, some studies have shown that the expression of FTO is related to the occurrence and prognosis of gastric cancer. Experimental data have shown that FTO in a low expression state has the ability to inhibit the proliferation, migration, and invasion of gastric cancer. However, high expression of FTO exerts an opposite result, which promotes the activity of gastric cancer cells (Xu et al., 2017). FTO also provided deep insights in AML (Li Z. et al., 2017; Huang et al., 2019), breast cancer (Niu et al., 2019; Xu et al., 2020), and melanoma (Yang et al., 2019).

**FIGURE 3:**
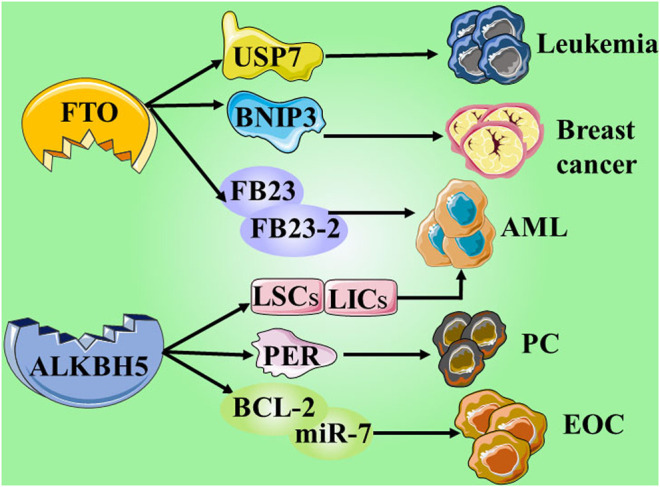
Give examples of the role and mechanisms involved erasers in cancers.

ALKBH5, another m6A demethylase of messenger RNA (mRNA) in higher eukaryotes, has also shown impressive performance. However, compared with the research on FTO, the research on ALKBH5 is still not thorough and abundant. In a study published in 2021, it was reported that the expression of ALKBH5 can be activated by Myc, and that the expression can reduce the m6A level in some selected Myc suppressor genes. Further experiments have also found that inhibiting the overexpression of ALKBH5 or selected Myc inhibitor genes can effectively inhibit the growth of Myc-mediated B-cell lymphoma *in vivo* and *in vitro* (Wu et al., 2021). In another study on osteosarcoma, ALKBH5 was also found to inhibit the occurrence and development of tumors through m6A-dependent epigenetic silencing of the pre-miR-181b-1/YAP signal axis present in osteosarcoma (Yuan et al., 2021). It is worth mentioning a recent novel discovery that ALKBH5 is not involved in DNA repair, but studies have found that demethylation of ALKBH5 may play a supporting role in maintaining genome integrity. In terms of the mechanism, the overexpression of ALKBH5 reduces the level of 3-methylcytosine in genomic DNA and the cytotoxic effect of the DNA-damaging alkylating agent methyl methanesulfonate (Akula et al., 2021) (Table 1 and Figure 3; ; Fujii et al., 2013; Li et al., 2015; Barbieri et al., 2017; Karakashev et al., 2018; Ma et al., 2019; Chen et al. (2019); Li et al. (2019b); Niu et al. (2019); Zhu et al., 2019; Li et al. (2019a); Zou et al. (2019); Yang et al., 2020; Chen et al., 2020b; Li et al., 2020; Chen et al., 2020a; Huang et al., 2019; Su et al., 2020; Guo et al., 2020; Shen et al., 2020; Seo et al., 2021)

## Interaction

Epigenetic modification is not a static entity, but it develops in a way dependent on the cellular environment and dynamically changes to cope with a complex environment. It can be simply understood as follows: epigenetic modifications undergo dynamic changes. Under a certain physiological environment, certain modifications will be added, and when external factors or physiological environments change, these newly added modifications will be removed. As mentioned earlier, epigenetic writers and erasers have proven to be ubiquitous in cancer, and epigenetic changes at different types and sites usually represent different meanings. This also provides many potential targets for cancer research and treatment. A large number of small molecule drugs are currently being developed mainly to target these epigenetic regulatory factors. Although only a few epigenetic drugs have been certified by the FDA for cancer treatment, a large number of excellent epigenetic drugs have gone through clinical research on cancer treatment and have achieved remarkable results (Ganesan et al., 2019). The above discussion has already been involved. However, there is no research linking epigenetic drugs and cancer treatment to prove whether there is a certain synergy. This point will be elaborated subsequently.

### Enhancement of Chemotherapy Sensitivity

Chemotherapy is one of the most important methods for the treatment of malignant tumors. However, the resistance of tumor cells to chemotherapeutic drugs often leads to failure of chemotherapy. Therefore, it is particularly important to resensitize the patients to chemotherapy-resistant drugs. Among the existing writers, DNMT decitabine can resensitize the patients with ovarian cancer to platinum-based drugs, and its mechanism may involve demethylation and re-expression in DNA repair and immune activation pathways (Fang et al., 2018). Sara et al. also strongly agreed with this finding and believed that epigenetic therapy has great potential in ovarian cancer (Moufarrij et al., 2019).

In breast cancer, Ye et al. found that the restoration of Spalt-like transcription factor 2 (SALL2) is mediated by DNMT inhibition, which can make tamoxifen-resistant breast cancer sensitive to tamoxifen therapy again. This discovery reveals and represents a potential clinical target that can be used for the treatment of tamoxifen-resistant breast cancer. These patients can benefit from the combination therapy of tamoxifen and DNMT inhibitors (Ye et al., 2019).

In colorectal cancer, Wei et al. confirmed that zebularine, a DNMT inhibitor with low toxicity, overcomes hypoxia-induced resistance to oxaliplatin in HCT116 cells by down-regulating the expression of hypoxia-inducible factor-1α (HIF-1α). It also provides a new strategy for the treatment of colorectal cancer, which is to overcome the resistance to oxaliplatin by increasing the hydroxylation of HIF-1α (Wei et al., 2020).

Similarly, in gefitinib-resistant PC9/AB2 cells, the combination of the EZH2 inhibitor GSK343 and gefitinib could significantly inhibit the activity of drug-resistant cells, reduce the migration ability of the cells, and induce the apoptosis of drug-resistant cells (Gong et al., 2019). EZH2 also showed surprising results in reversing the chemotherapy resistance of cervical cancer cells (Cai et al., 2016), cisplatin resistance in epithelial ovarian cancer (Liu et al., 2014), melanoma resistance to immunotherapy (Emran et al., 2019), and resistance of prostate cancer (Shankar et al., 2020), and it can be described as the gospel for patients.

Not only the writer, but the eraser is also excellent at improving the resistance to chemicals (Xiang et al., 2020). Zhou et al. demonstrated for the first time that FTO with elevated gene levels in cervical squamous cell carcinoma (CSCC) tissues can enhance the resistance of CSCC to radiotherapy and chemotherapy. The mechanism may be upregulation of β-catenin and subsequent activation of excision repair cross-complementation group 1 (ERCC1), and ultimately it was attributed to the demethylation of FTO (Zhou et al., 2018a). According to a report in 2020, malignant glioma is one of the deadly primary brain tumors in adults. Li et al. found that the inhibition of FTO can enhance the anti-tumor effect of the chemotherapeutic drug temozolomide in malignant glioma, and its mechanism may involve the MYC-miR-155/23a cluster-MXI1 feedback loop (Xiao et al., 2020). Compared with the well-researched FTO, there are relatively few studies on the resistance of ALKBH. Alkylating agents have broad-spectrum effects. It has been reported that it has anti-tumor, immunosuppressive, and other therapeutic effects, as well as carcinogenic, teratogenic, mutagenic, and bone marrow suppressive effects. Although alkylating agents have high toxicity and carcinogenic potential, they are still important first-line anti-tumor drugs for many highly aggressive and metastatic cancers (Sauter and Gillingham, 2020). Tran et al. inhibited the DNA repair activity of the ALKBH enzyme by targeting glutamine metabolism, leading to the accumulation of DNA alkylation damage, thereby increasing the sensitivity of cells to alkylating agents (Tran et al., 2017). The combination of drugs has achieved the effect of improving the efficacy and reducing the side effects of alkylating agents, which can be considered as a new strategy. In addition, the glutaminase inhibitor CB-839 has entered clinical trials for determining its safety and activity (Riess et al., 2021).

### Inhibition of Proliferation and Promotion of Apoptosis

As a DNMT inhibitor, RG108 has been proven to effectively inhibit the proliferation of endometrial cancer cells and block the G2/M phase of the cell cycle to induce apoptosis, and it can be regarded as a promising drug candidate for endometrial cancer patients (Yang et al., 2017). Coincidentally, zebularine, another DNMT inhibitor, is thought to induce apoptosis in gastric cancer cells through the mitochondrial pathway (Tan et al., 2013). In addition, zebularine has also been shown to inhibit the proliferation of lung cancer A549 cells and HeLa cervical cancer cells through cell cycle arrest and apoptosis (You and Park, 2012; You and Park, 2014). Zhang et al. also stated that the combined application of DNMT and mTOR signals can inhibit the formation and proliferation of colorectal cancer (Zhang et al., 2009).

Kazuya Ishiguro et al. discussed the role of DOT1L in the development of multiple myeloma, and they believed that the inhibitory effect of DOT1L may be a new therapy for myeloma. Further experiments showed that the use of DOT1L inhibitors in multiple myeloma can induce cell cycle arrest and apoptosis, and can strongly inhibit cell proliferation *in vitro* (Ishiguro et al., 2019). Li et al. explored the effects of DOT1L inhibitor EPZ-5676 in combination with chemotherapy drugs on the proliferation and apoptosis of human ALL cells, and they concluded that the combination of these drugs has a synergistic inhibitory effect on proliferation, and low concentrations of EPZ-5676 combined with different chemotherapy drugs cause synergistic induction of apoptosis and have pro-apoptotic effects (Li LH. et al., 2017). However, the achievements of EZH2 in inhibiting proliferation and promoting apoptosis are countless, and they have been observed in colorectal cancer (Xu et al., 2018; Li X. et al., 2019), breast cancer (Han et al., 2018), and bladder cancer (Chen et al., 2019).

Similarly, FTO and ALKBH, the only known erasers, also have great potential in inhibiting proliferation and promoting apoptosis. In the study by Tang et al., it was suggested that FTO is necessary for the proliferation of pancreatic cancer cells by using RNA interference and knocking out FTO. With respect to its basic mechanism, Tang et al. believed that FTO is related to the interaction of MYC proto-oncogene and BHLH transcription factor (Tang et al., 2019). In the review of FTO by Wang et al., they believed that FTO is involved in a variety of biological processes, such as cancer cell apoptosis, proliferation, migration, metastasis, and stem cell self-renewal in human cancer. These modulations mainly rely on the communication between FTO and specific signal pathways, including PI3K/AKT, MAPK and mTOR signal pathways (Wang et al., 2020).

With respect to another type of eraser, ALKBH5, studies have found that silencing of ALKBH5 can significantly increase the proliferation, migration, and invasion of pancreatic cancer cells, and its overexpression will cause the opposite effect (Tang et al., 2020). In the study by Jin et al., it is believed that effective inhibition of the m6A modification of ALKBH5 may constitute a potential treatment strategy for lung cancer. Further experiments showed that ALKBH5 inhibited tumor proliferation and metastasis by reducing YTHDFs-mediated YAP expression and inhibiting miR-107/LATS2-mediated YAP activity in NSCLC (Jin et al., 2020). In general, both the writer and the eraser have their own unique advantages, attracting the attention of researchers.

## Conclusion

Epigenetic modification is becoming a significant topic for research. With the development of next-generation sequencing technology, it will be possible explore more unknown areas for the world. As an important part of it. The resulting drugs that target epigenetic modifications will also be closely related to diseases, especially cancer. The change of epigenetic factors in cancer provides new targets for the development of cancer drugs, not only limited to a single therapy, but combined drugs that can achieve the desired efficacy. As with the emergence of any other new therapies, the fever of epigenetic modification is also essential to bring about adverse reactions. In the study of Allen et al., epigenetics affects gene expression, which may lead to adverse reactions of statins and have long-term effects on the health of statin users (Allen and Mamotte, 2017). In addition, the general public’s attitude toward new therapies is not very positive. Paradoxically, any new thing is also pursued by the public. In the endless research on epigenetic modification, a large number of options will bring the dawn on cancer and even more refractory diseases.
