# Supplementary material for: Methylation Landscape: Targeting Writer or Eraser to Discover Anti-Cancer Drug
Source: Front Pharmacol. 2021 Jun 3;12:690057. doi: 10.3389/fphar.2021.690057 (PMC8209422; doi:10.3389/fphar.2021.690057)
Supplement: Supplementary file 1 [file Table1.DOCX]

Table 1 Describe the author and eraser mechanism in cancer.

| **Component** | **Disease** | **Target** | **Function** | **Regulation** | **Refs** |
| --- | --- | --- | --- | --- | --- |
| METTL3 | AML | Promoter | Writers | Downregulation of it leads to cell cycle arrest and leukemia cell differentiation | (Barbieri et al., 2017) |
| METTL3 | CRC | SOX2 | Writers | Promote the progression of CRC through m6A-IGF2BP2-dependent mechanism | (Li et al., 2019b) |
| METTL14 | CRC | XIST | Writers | Inhibit the proliferation and metastasis of CRC by down-regulating XIST | (Yang et al., 2020) |
| METTL14 | CRC | SOX4 | Writers | Inhibit the migration, invasion and metastasis of CRC cells through SOX4 | (Chen et al., 2020b) |
| WTAP | HCC | ETS1 | Writers | Promotes the progression of HCC through m6A-HuR-dependent epigenetic silencing of ETS1 | (Chen et al., 2019) |
| DNMT | CML | MNC bone marrow | Writers | The expression level of DNMT mRNA is related to the disease progression of CML. | (Li et al., 2015) |
| DNMT1 | GC | PCDH10 | Writers | By interacting with HOTAIR and miR-148b, it leads to the methylation of PCDH10, thereby promoting the development of GC | (Seo et al., 2021) |
| DNMT1 | HCC | miR-148a-3p | Writers | Block the negative regulation between miR-148a-3p, thereby inhibiting the stem cell characteristics of HCC cells. | (Li et al., 2020) |
| EZH2 | PTEN-mutated cancer | FOXO1 | Writers | Inhibition of FOXO1 can treat PTEN-proficient cancers | (Ma et al., 2019) |
| EZH2 | EOC | PRMT4 | Writers | The activity of EZH2 determines the level of PRMT4 expression in EOC | (Karakashev et al., 2018) |
| WTAP | SaOS | HMBOX1 | Writers | Inhibit the expression of HMBOX1 in an m6A-dependent manner to promote the occurrence of SaOS. | (Chen et al., 2020a) |
| FTO | AML | FB23  FB23-2 | Erasers | Inhibit proliferation and promote differentiation/apoptosis of AML cell lines | (Huang et al., 2019) |
| FTO | Leuke-  mia | LILRB4 | Erasers | Inhibit the maintenance and immune escape of cancer stem cells | (Su et al., 2020) |
| FTO | NSCLC | USP7 | Erasers | Promote the growth of NSCLC by regulating the m6A level of USP7 mRNA | (Li et al., 2019a) |
| FTO | Cervical cancer | E2F1  Myc | Erasers | Overexpression of E2F1 or Myc can make up for the lack of FTO, thereby reducing cell proliferation and migration | (Zou et al., 2019) |
| FTO | Breast tumor | BNIP3 | Erasers | Promote breast tumor progression by inhibiting BNIP3 | (Niu et al., 2019) |
| ALKBH2 | Bladder cancer | MUC1 | Erasers | Promote the development of bladder cancer by regulating the expression of MUC1 | (Fujii et al., 2013) |
| ALKBH5 | EOC | MiR-7  BCL-2 | Erasers | Inhibition of autophagy in epithelial ovarian cancer through miR-7 and BCL-2 | (Zhu et al., 2019) |
| ALKBH5 | PC | PER1 | Erasers | Prevents the progression of pancreatic cancer through the post-transcriptional activation of PER1 that depends on m6A-YTHDF2 | (Guo et al., 2020) |
| ALKBH5 | AML | LSCs  LICs | Erasers | Selectively promote tumorigenesis and cancer stem cell self-renewal in AML | (Shen et al., 2020) |

METTL3: methyltransferase-like 3; CRC: Colorectal carcinoma; SOX2: sex determining region Y-box 2; XIST:X inactivate-specific transcript; SOX4: SRY-related high-mobility-group box; WTAP: Wilms tumor 1-associated protein; HCC: hepatocellular carcinoma; ETS1: ETS proto-oncogene 1; SaOS: osteosarcoma; CML: Chronic Myeloid Leukemia; PCDH10: Protocadherin 10; FOXO1: Forkhead box transcription factor-1; PRMT: protein arginine methyltransferase; EOC: Epithelial ovarian cancer; NSCLC: non-small cell lung cancer; Erasers: ubiquitin-specific protease; EOC: epithelial ovarian cancer; LSCs/LICs: leukemia stem/initiating cells.

Figure 1. Methylation interactions between writers and erasers.

Figure 2. Give examples of the role and mechanisms involved writers in cancers.

Figure 3. Give examples of the role and mechanisms involved erasers in cancers.

Barbieri, I., Tzelepis, K., Pandolfini, L., Shi, J., Millán-Zambrano, G., Robson, S.C., Aspris, D., Migliori, V., Bannister, A.J., Han, N., De Braekeleer, E., Ponstingl, H., Hendrick, A., Vakoc, C.R., Vassiliou, G.S., Kouzarides, T., 2017. Promoter-bound METTL3 maintains myeloid leukaemia by m(6)A-dependent translation control. Nature 552, 126-131. 10.1038/nature24678

Chen, S., Li, Y., Zhi, S., Ding, Z., Wang, W., Peng, Y., Huang, Y., Zheng, R., Yu, H., Wang, J., Hu, M., Miao, J., Li, J., 2020a. WTAP promotes osteosarcoma tumorigenesis by repressing HMBOX1 expression in an m(6)A-dependent manner. Cell Death Dis 11, 659. 10.1038/s41419-020-02847-6

Chen, X., Xu, M., Xu, X., Zeng, K., Liu, X., Pan, B., Li, C., Sun, L., Qin, J., Xu, T., He, B., Pan, Y., Sun, H., Wang, S., 2020b. METTL14-mediated N6-methyladenosine modification of SOX4 mRNA inhibits tumor metastasis in colorectal cancer. Mol Cancer 19, 106. 10.1186/s12943-020-01220-7

Chen, Y., Peng, C., Chen, J., Chen, D., Yang, B., He, B., Hu, W., Zhang, Y., Liu, H., Dai, L., Xie, H., Zhou, L., Wu, J., Zheng, S., 2019. WTAP facilitates progression of hepatocellular carcinoma via m6A-HuR-dependent epigenetic silencing of ETS1. Mol Cancer 18, 127. 10.1186/s12943-019-1053-8

Fujii, T., Shimada, K., Anai, S., Fujimoto, K., Konishi, N., 2013. ALKBH2, a novel AlkB homologue, contributes to human bladder cancer progression by regulating MUC1 expression. Cancer Sci 104, 321-327. 10.1111/cas.12089

Guo, X., Li, K., Jiang, W., Hu, Y., Xiao, W., Huang, Y., Feng, Y., Pan, Q., Wan, R., 2020. RNA demethylase ALKBH5 prevents pancreatic cancer progression by posttranscriptional activation of PER1 in an m6A-YTHDF2-dependent manner. Mol Cancer 19, 91. 10.1186/s12943-020-01158-w

Huang, Y., Su, R., Sheng, Y., Dong, L., Dong, Z., Xu, H., Ni, T., Zhang, Z.S., Zhang, T., Li, C., Han, L., Zhu, Z., Lian, F., Wei, J., Deng, Q., Wang, Y., Wunderlich, M., Gao, Z., Pan, G., Zhong, D., Zhou, H., Zhang, N., Gan, J., Jiang, H., Mulloy, J.C., Qian, Z., Chen, J., Yang, C.G., 2019. Small-Molecule Targeting of Oncogenic FTO Demethylase in Acute Myeloid Leukemia. Cancer Cell 35, 677-691.e610. 10.1016/j.ccell.2019.03.006

Karakashev, S., Zhu, H., Wu, S., Yokoyama, Y., Bitler, B.G., Park, P.H., Lee, J.H., Kossenkov, A.V., Gaonkar, K.S., Yan, H., Drapkin, R., Conejo-Garcia, J.R., Speicher, D.W., Ordog, T., Zhang, R., 2018. CARM1-expressing ovarian cancer depends on the histone methyltransferase EZH2 activity. Nat Commun 9, 631. 10.1038/s41467-018-03031-3

Li, J., Han, Y., Zhang, H., Qian, Z., Jia, W., Gao, Y., Zheng, H., Li, B., 2019a. The m6A demethylase FTO promotes the growth of lung cancer cells by regulating the m6A level of USP7 mRNA. Biochem Biophys Res Commun 512, 479-485. 10.1016/j.bbrc.2019.03.093

Li, T., Hu, P.S., Zuo, Z., Lin, J.F., Li, X., Wu, Q.N., Chen, Z.H., Zeng, Z.L., Wang, F., Zheng, J., Chen, D., Li, B., Kang, T.B., Xie, D., Lin, D., Ju, H.Q., Xu, R.H., 2019b. METTL3 facilitates tumor progression via an m(6)A-IGF2BP2-dependent mechanism in colorectal carcinoma. Mol Cancer 18, 112. 10.1186/s12943-019-1038-7

Li, X., Wang, L., Cao, X., Zhou, L., Xu, C., Cui, Y., Qiu, Y., Cao, J., 2020. Casticin inhibits stemness of hepatocellular carcinoma cells via disrupting the reciprocal negative regulation between DNMT1 and miR-148a-3p. Toxicol Appl Pharmacol 396, 114998. 10.1016/j.taap.2020.114998

Li, Y.H., Liu, X.D., Guo, X.F., Liu, X., Luo, J.M., Zhang, Y.X., Li, Z.S., 2015. [Expression and Clinical Significance of DNMT in Patients with Chronic Myeloid Leukemia]. Zhongguo Shi Yan Xue Ye Xue Za Zhi 23, 1547-1550. 10.7534/j.issn.1009-2137.2015.06.003

Ma, L., Yan, Y., Bai, Y., Yang, Y., Pan, Y., Gang, X., Karnes, R.J., Zhang, J., Lv, Q., Wu, Q., Huang, H., 2019. Overcoming EZH2 Inhibitor Resistance by Taxane in PTEN-Mutated Cancer. Theranostics 9, 5020-5034. 10.7150/thno.34700

Niu, Y., Lin, Z., Wan, A., Chen, H., Liang, H., Sun, L., Wang, Y., Li, X., Xiong, X.F., Wei, B., Wu, X., Wan, G., 2019. RNA N6-methyladenosine demethylase FTO promotes breast tumor progression through inhibiting BNIP3. Mol Cancer 18, 46. 10.1186/s12943-019-1004-4

Seo, S.I., Yoon, J.H., Byun, H.J., Lee, S.K., 2021. HOTAIR Induces Methylation of PCDH10, a Tumor Suppressor Gene, by Regulating DNMT1 and Sponging with miR-148b in Gastric Adenocarcinoma. Yonsei Med J 62, 118-128. 10.3349/ymj.2021.62.2.118

Shen, C., Sheng, Y., Zhu, A.C., Robinson, S., Jiang, X., Dong, L., Chen, H., Su, R., Yin, Z., Li, W., Deng, X., Chen, Y., Hu, Y.C., Weng, H., Huang, H., Prince, E., Cogle, C.R., Sun, M., Zhang, B., Chen, C.W., Marcucci, G., He, C., Qian, Z., Chen, J., 2020. RNA Demethylase ALKBH5 Selectively Promotes Tumorigenesis and Cancer Stem Cell Self-Renewal in Acute Myeloid Leukemia. Cell Stem Cell 27, 64-80.e69. 10.1016/j.stem.2020.04.009

Su, R., Dong, L., Li, Y., Gao, M., Han, L., Wunderlich, M., Deng, X., Li, H., Huang, Y., Gao, L., Li, C., Zhao, Z., Robinson, S., Tan, B., Qing, Y., Qin, X., Prince, E., Xie, J., Qin, H., Li, W., Shen, C., Sun, J., Kulkarni, P., Weng, H., Huang, H., Chen, Z., Zhang, B., Wu, X., Olsen, M.J., Müschen, M., Marcucci, G., Salgia, R., Li, L., Fathi, A.T., Li, Z., Mulloy, J.C., Wei, M., Horne, D., Chen, J., 2020. Targeting FTO Suppresses Cancer Stem Cell Maintenance and Immune Evasion. Cancer Cell 38, 79-96.e11. 10.1016/j.ccell.2020.04.017

Yang, X., Zhang, S., He, C., Xue, P., Zhang, L., He, Z., Zang, L., Feng, B., Sun, J., Zheng, M., 2020. METTL14 suppresses proliferation and metastasis of colorectal cancer by down-regulating oncogenic long non-coding RNA XIST. Mol Cancer 19, 46. 10.1186/s12943-020-1146-4

Zhu, H., Gan, X., Jiang, X., Diao, S., Wu, H., Hu, J., 2019. ALKBH5 inhibited autophagy of epithelial ovarian cancer through miR-7 and BCL-2. J Exp Clin Cancer Res 38, 163. 10.1186/s13046-019-1159-2

Zou, D., Dong, L., Li, C., Yin, Z., Rao, S., Zhou, Q., 2019. The m(6)A eraser FTO facilitates proliferation and migration of human cervical cancer cells. Cancer Cell Int 19, 321. 10.1186/s12935-019-1045-1
