# Supplementary material for: Methylation Landscape: Targeting Writer or Eraser to Discover Anti-Cancer Drug
Source: Front Pharmacol. 2021 Jun 3;12:690057. doi: 10.3389/fphar.2021.690057 (PMC8209422; doi:10.3389/fphar.2021.690057)
Supplement: Supplementary file 2 [file Table2.DOCX]

| Drugs | **Target** | Regulation |
| --- | --- | --- |
| zazcytidine | DNMT | inhibitors of DNA methylation |
| decitabine | DNMT | inhibitors of DNA methylation |
| Tazemetostat | Ezh2 | inhibitors of histone methylation |
| Pinometostat | DOT1L | inhibitors of histone methylation |
| EPZ004777 | DOT1L | inhibitors of histone methylation |
| PsA-3091 | DOT1L | inhibitors of histone methylation |
| Rhein | FTO | inhibitors of N^6^ -methyladenosine (m^6^ A) demethylase |
| Meclofenamic acid | FTO | inhibitors of N^6^ -methyladenosine (m^6^ A) demethylase |
| MO-I-500 | FTO | inhibitors of N^6^ -methyladenosine (m^6^ A) demethylase |
| fluorescein | FTO | inhibitors of N^6^ -methyladenosine (m^6^ A) demethylase |
| R-2-hydroxyglutarate | FTO | inhibitors of N^6^ -methyladenosine (m^6^ A) demethylase |
| CS1 | FTO | inhibitors of N^6^ -methyladenosine (m^6^ A) demethylase |
| CS2 | FTO | inhibitors of N^6^ -methyladenosine (m^6^ A) demethylase |
| Zebularine | DNMT | inhibitors of DNA methylation |
| RG108 | DNMT | inhibitors of DNA methylation |
| zebularine | DNMT | inhibitors of DNA methylation |
| EPZ-5676 | DOT1L | inhibitors of histone methylation |
